# ^18^F-fluorocholine PET/CT in patients with occult biochemical recurrence of prostate cancer: Detection rate, impact on management and adequacy of impact. A prospective multicentre study

**DOI:** 10.1371/journal.pone.0191487

**Published:** 2018-02-09

**Authors:** Quentin Gillebert, Virginie Huchet, Caroline Rousseau, Alexandre Cochet, Pierre Olivier, Frédéric Courbon, Eric Gontier, Valérie Nataf, Sona Balogova, Jean-Noël Talbot, the other ICHOROPRO investigators

**Affiliations:** 1 Department of Nuclear Medicine, Hôpital Tenon, Assistance Publique-Hôpitaux de Paris & Université Pierre et Marie Curie, Paris, France; 2 Nuclear Medicine Unit, ICO Cancer Center, Saint Herblain, France; 3 Department of Nuclear Medicine, Centre GF Leclerc, Dijon, France; 4 Department of Nuclear Medicine, CHU Brabois, Vandoeuvre-lès-Nancy, France; 5 Department of Nuclear Medicine, Institut Claudius Regaud, Toulouse, France; 6 Department of Nuclear Medicine, HIA Val-de-Grâce, Paris, France; 7 Department of radiopharmacy, Hôpital Tenon, Assistance Publique-Hôpitaux de Paris, Paris, France; 8 Department of Nuclear Medicine, Comenius University & St Elisabeth Oncology Institute, Bratislava, Slovakia; Wayne State University, UNITED STATES

## Abstract

**Aim:**

To prospectively evaluate the clinical impact and the diagnostic performance of FCH-PET/CT in patients with occult biochemical recurrence of prostate cancer (PCa).

**Materials and methods:**

Results of 179 patients (mean PSA = 7.5ng/mL) with negative/inconclusive results of pelvic-MRI and of bone-scintigraphy were analysed. To determine the impact of FCH-PET/CT on diagnostic thinking and on patient management, the referring physicians prospectively filled-in a 1^st^ and 2^nd^ questionnaire related to patient’s planned management before and after FCH-PET/CT. Based on data from a 6-month follow-up after FCH-PET/CT, an independent assessor blinded to results of FCH-PET/CT determined the adequacy of management changes motivated by FCH-PET/CT.

**Results:**

FCH-PET/CT localised foci evocative of recurrent PCa in 59% (105/179) of patients. Results of FCH-PET/CT motivated a change in scheduled patient management in 56% (100/179) of patients; which was considered as adequate in 89% (89/100) of patients. FCH-PET/CT also led to the detection of lung cancer in two patients.

**Conclusion:**

FCH PET/CT is a powerful tool to localise the sites of occult biochemical recurrence of PCa, leading to an adequate management change in half of patients.

## Introduction

Prostate cancer (PCa) is the most common malignancy and the second leading cause of death in men in Europe and North America [1]. Biochemical recurrence defined by an increase of prostate specific antigen (PSA) serum level above 0.2ng/mL after radical prostatectomy (RP) or nadir value +2ng/mL after external beam radiotherapy (EBRT) occurs in 27 to 53% of PCa patients within 10 years after initial treatment with curative intent, like radical prostatectomy RP or EBRT [2].

Local salvage treatment of recurrent PCa can be indicated in the absence of metastatic lymph node involvement, distant metastasis or second primary cancer. A whole-body imaging method with reliable diagnostic accuracy is thus needed.

Computed tomography (CT) of abdomen and pelvis or bone scintigraphy (BS) show a limited sensitivity for early localisation of PCa recurrence and most likely yield a negative result when serum PSA serum level are less than 20ng/mL [2–4].

Magnetic resonance imaging (MRI) of the pelvis has been shown to be a reliable tool in the evaluation of the prostatic bed [2, 5, 6]; ^18^F-fluoromethyl-dimethyl-2-hydroxyethylammonium (^18^F-fluorocholine or FCH) is approved in several EU countries for functional imaging of PCa lesions.

The usefulness of FCH Positron Emission Tomography–Computed Tomography (PET/CT) in the localisation of PCa foci in patients with rising serum PSA level after treatment has been extensively documented [3, 7–10] and FCH PET/CT can also be helpful in guiding salvage radiation therapy [11, 12]. Available results of retrospective single centre studies also suggest that FCH PET/CT could have a significant clinical impact on PCa patient management in case of biochemical recurrence [13–15].

We report the results of the first prospective multicentre clinical study evaluating the impact of FCH PET/CT on patient management and the adequacy of the therapeutic decisions motivated by FCH PET/CT, in patients with occult biochemical recurrence of PCa.

## Materials and methods

### Patients

Patients with biochemical recurrence of PCa after treatment with curative intent (RP, EBRT, high intensity focused ultrasounds—HIFU or brachytherapy) were prospectively included in the ICHOROPRO study (Eudract 2007-004419-69).

The main inclusion criteria were as follows: PSA serum level >2ng/mL or >1ng/mL with a doubling time <3 months or >0.5ng/mL with a Gleason score ≥7 and a doubling time <6 months. PSA serum level was determined within 3 months before FCH PET/CT. The results of conventional imaging modalities, at least recent pelvis MRI and BS, must have been negative or equivocal.

The exclusion criteria were: radiotherapy within 4 months, other known active malignancy or inflammatory disorder and any change in PCa treatment that occurred after the last MRI, BS or serum PSA assay.

### FCH PET/CT acquisition protocol

Imaging was performed on hybrid PET/CT cameras (Discovery, GE Healthcare; Gemini, Philips or Biograph, Siemens). All patients were fasting for at least 6 hours before intravenous injection of 3 to 4 MBq of FCH (IASOCholine, IASON GmbH) per kg of body mass.

The dynamic image acquisition was centred on the pelvic region and started immediately after administration of FCH: a low dose CT for attenuation correction and anatomic localisation of lesions was performed and followed by an 8 minute list-mode PET acquisition (1min/frame). Then a whole-body low dose CT from the skull to mid-thigh followed by whole-body PET acquisition was performed.

### Impact of FCH PET/CT and adequacy of changes in patient management

The referring physicians prospectively filled in two questionnaires concerning the patient’s therapeutic management; in the first one, filled at patient inclusion, the scheduled treatment before FCH PET/CT was recorded and, in the second one, the management strategy taking into account the report of the FCH PET/CT was described (the example of second questionnaire is provided in S1 Table). FCH PET/CT was considered to have an impact on patient management if the modification of therapeutic plan was motivated by the result of FCH PET/CT. FCH PET/CT was considered to have an impact on diagnostic thinking if the result of FCH PET/CT prompted complementary diagnostic tests (e.g. biopsy, CT or MRI).

The adequacy of changes in management motivated by FCH PET/CT was evaluated by an independent assessor in view of all follow-up data in case of modification of reported impact of FCH PET/CT on decision making.

### Statistics

The non-parametric Mann-Whitney or Wilcoxon tests were used to compare unpaired or paired quantitative data, respectively. A probability level p<0.05 was considered significant.

## Results

Between July 2008 and October 2009, 181 patients met the inclusion criteria, gave their written informed consent and underwent FCH PET/CT, in one of the fifteen centres involved in the study. Patients were then followed during a minimum of 6 months after FCH PET/CT, except for 2 patients who were lost to follow-up after a shorter period of time.

Overall, the results of 179 patients (mean 13, median 7, range 2–54 per centre) from fourteen centres (Angers n = 2, Bordeaux n = 8, Dijon n = 19, Lyon n = 5, Marseille n = 4, Nancy n = 11, Nantes n = 40, Nice n = 2, Nîmes n = 3, Paris (Hospital Tenon) n = 55, Paris (Hospital Val-de-Grâce) n = 8, Rennes n = 6, Rouen n = 6, Toulouse n = 10) were analysed whose characteristics are shown in Table 1. Forty-eight patients were in their second biochemical recurrence of PCa, the first recurrence having been treated by radiotherapy, ADT or HIFU.

**Table 1:** Characteristics of the 179 patients.

Parameters	Data
**Age of the patients (years) mean ± SD**	69 ±7.3
median (range)	69 (49–87)
**PSA serum level at FCH PET/CT (ng/mL)**	
mean ± SD	7.5 ± 19.1
median (range)	3.6 (0.5–244)
**Gleason score**	
median (range) distribution	7 (2–9)
<6	16
6	43
7	90
8	23
>8	3
Unknown	4
**Initial treatment**	
Radical prostatectomy	73
Radiotherapy	62
Both prostatectomy and radiotherapy	19
Brachytherapy	15
HIFU	7
Transurethral resection of the prostate	3
**Previous ADT**	45
**Patients with 2**^**nd**^ **recurrence (pts.)**	48

### Detection rate of FCH PET/CT and relation with PSA serum level

**Fig 1:**
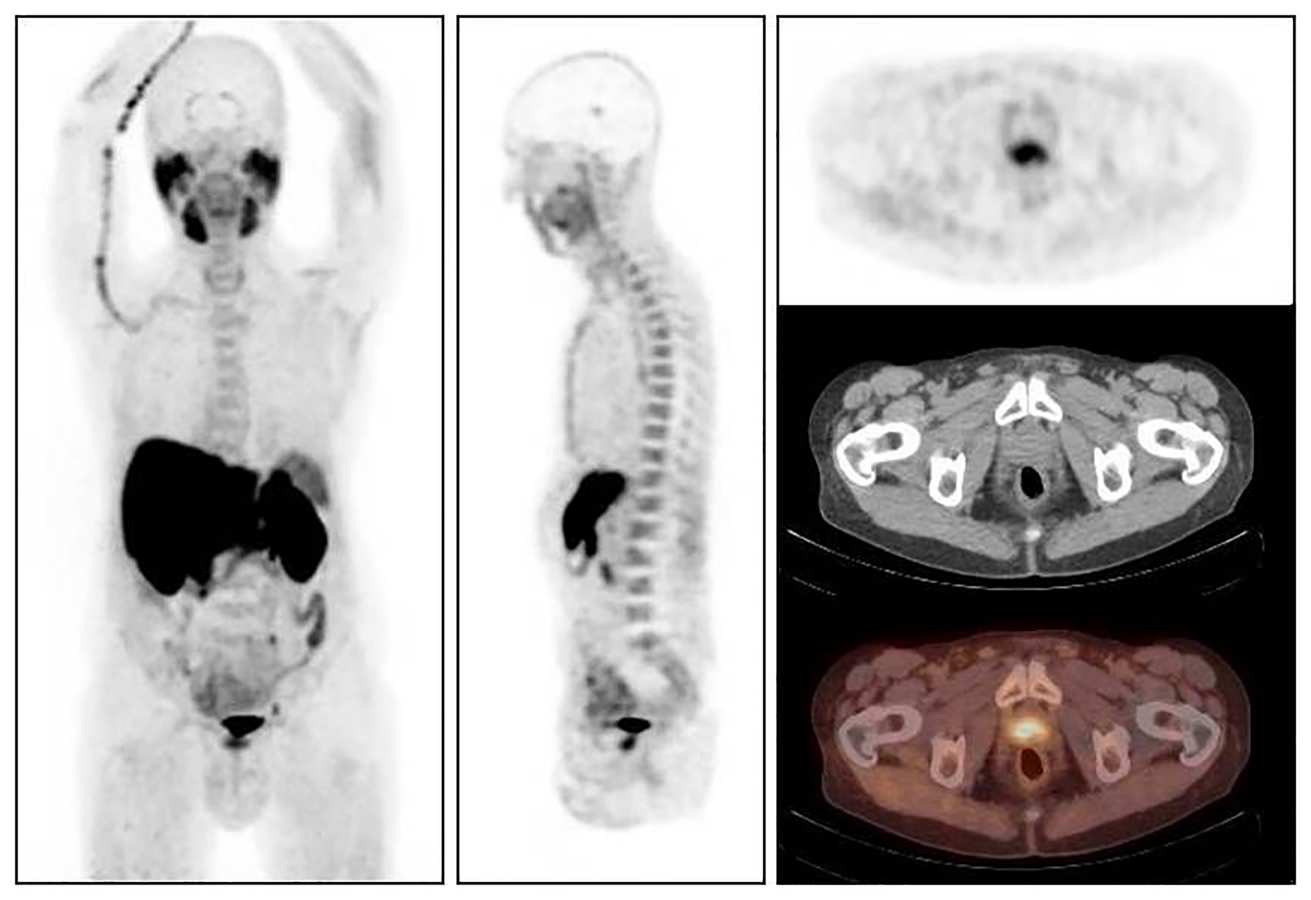
76y old patient with prostate cancer Gleason score 7, treated 5 years ago by high intensity focused ultrasound (HIFU). Serum PSA level at the time of FCH PET/CT 9.1ng/mL. FCH PET/CT revealed isolated FCH uptake by two lobes of prostate but no foci evocative of regional of distant prostate cancer recurrence. Scheduled palliative medical treatment was replaced by radiation therapy with curative intent. Adequacy of this therapeutic decision was confirmed by clinical follow-up, serum PSA level 11 months after FCH PET/CT = 0.8 ng/mL.

**Fig 2:**
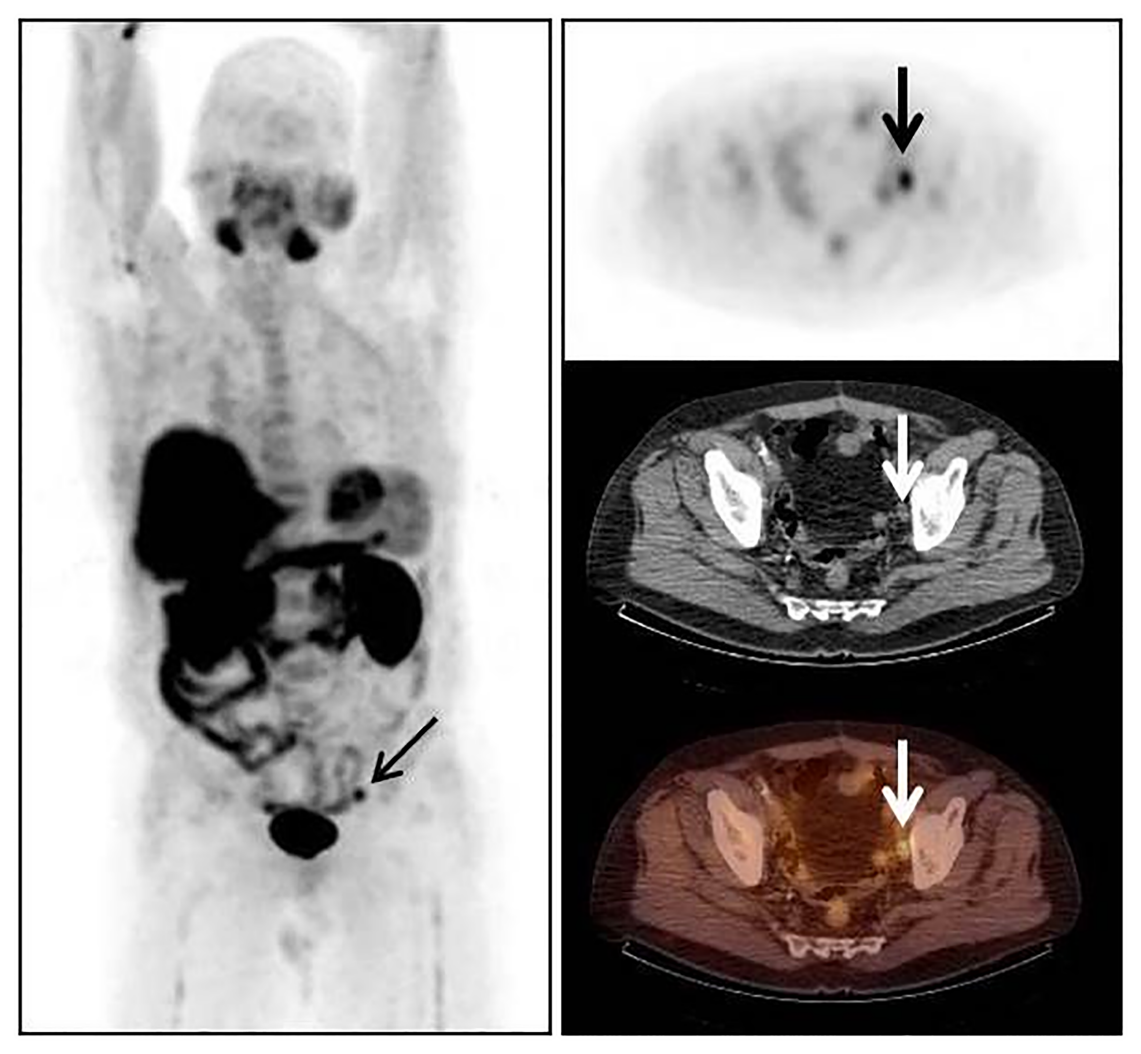
64y old patient with prostate cancer Gleason score 8 treated 6 years ago by radical prostatectomy. PSA serum level at the time of FCH PET/CT 3.5 ng/mL. Intense FCH uptake by left external iliac lymph node evocative of prostate cancer recurrence. Scheduled watchful waiting replaced by complete androgen blockade leading to complete remission of disease according to follow-up data.

**Fig 3:**
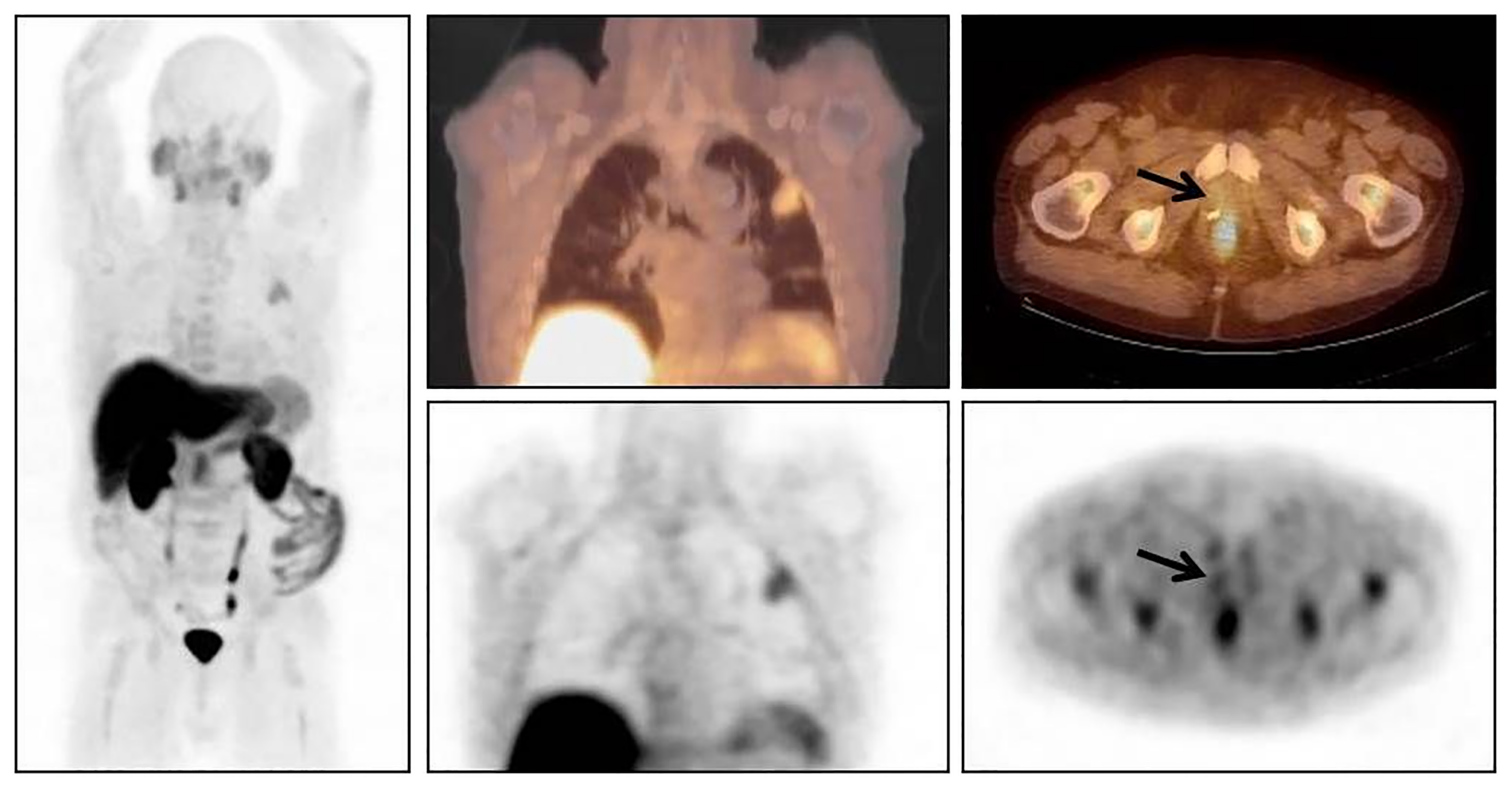
68y old patient with prostate cancer Gleason score 7 treated 1 year ago by radical prostatectomy. Serum PSA level at the time of FCH PET/CT 2.4ng/mL; On FCH PET/CT equivocal focus in the right prostate loge and left pulmonary lesion evocative of malignancy. Scheduled androgen deprivation therapy was replaced by watchful waiting and 4 months later, by radiation therapy on prostate loge. Pulmonary lesion was resected and histology confirmed poorly differentiated large cell lung cancer with visceral pleural invasion.

On a per-patient basis, 105 FCH PET/CT were reported as positive (59%) (Figs 1–3), 20 as equivocal (11%) and 54 (30%) as negative. Results of on-site reading are summarized in Table 2.

**Table 2:** Results of reading of FCH PET/CT (L = local recurrence inside prostate bed, RLN = regional lymph nodes, D = distant metastases or second primaries).

Parameters	Number of patients
**Patient-based:**	
Positive	105 (59%, CI = 48–71%)
Equivocal	20 (11%, CI = 7–17%)
Negative	54 (30%, CI = 23–39%)
**Site with foci quoted as positive:**	
Local recurrence (L) only	43
Regional lymph nodes (RLN) only	35
Distant foci (D) only	13
L + RLN	2
RLN + D	6
L + D	2
L + RLN + D	4
**Distant foci quoted as positive:**	
Overall	25 patients
Bone	15
Lung	7
Distant lymph nodes	6
Liver	1

FCH PET foci quoted as positive corresponded to a local recurrence in 51 patients (28%), regional lymph node (RLN) in 47 patients (26%) and distant metastases in 25 patients (14%). Distant positive foci were most frequently observed in the skeleton (15 patients).

There were 2 unknown thyroid nodules among foci with mild intensity quoted 1 (benign); their nature was not further investigated.

Considering the whole series of 179 patients, there was a significant difference between the median PSA serum levels according to the result of FCH PET/CT, either positive (5 ng/mL, range 0.6–244) or non-positive i.e. negative or equivocal (2.85ng/mL, range 0.5–13.4; p = 0.0001). Median PSA velocity was also significantly higher in patients with positive than with non-positive FCH PET/CT (0.4ng/mL/y, range -0.4–18.2, vs. 0.2 ng/mL/y, range -0.9–3.7; p = 0.0003). In contrast, PSA doubling time and Gleason score were not statistically different whether or not FCH PET/CT was positive.

At FCH PET/CT 13/179 patients have been treated by ADT with mean PSA serum levels 26±65.9 ng/mL median 5 (range 1.2–244) ng/mL.

### Impact of FCH PET/CT on diagnostic thinking and therapeutic management

As a consequence of FCH PET/CT findings, complementary imaging was performed in 28 patients: 11 contrast-enhanced CTs (6 thoracic, 3 abdomino-pelvic, and 2 spinal) and 17 MRIs (7 of the prostate bed, 2 pelvic, 2 abdominal, 1 liver and 5 spine-MRI) were (re)-performed. The results of FCH PET/CT and of the complementary imaging examination were in agreement in 79% (22/28) of patients.

A total of 33 biopsies were performed after FCH PET/CT, including 25 biopsies of the prostatic bed. The results of FCH PET/CT and of histology were in agreement in 79% (26/33) of cases. Concerning visceral foci on FCH PET/CT, a histologic proof was obtained for lung metastasis in 2 patients and for 2 lung second cancers (Fig 3). FCH PET/CT yielded a false-positive result in 4 cases, and a false-negative result in 3 other cases.

Overall, an impact of FCH PET/CT on therapeutic management was observed in 100 patients (56%, CI = 45–68%) with 74 positive, 7 equivocal and 19 negative FCH PET/CT by patient-based on-site reading.

All evaluable patients have been referred by 90 different physicians who prospectively filled-in a 1^st^ and 2^nd^ questionnaire related to patient’s planned management before and after FCH-PET/CT. The details of scheduled and actual managements are given in Table 3. In summary, the most frequently scheduled treatment prior to FCH PET/CT was ADT (106 cases); in those patients, the impact rate was 52%, limited to a change in ADT in a 4% rate. Surgery or radiotherapy was scheduled in 17 patients; the impact rate was 82%, limited to a change in radiotherapy planning in 1 patient. Watchful waiting was scheduled in 51 patients; the impact rate was 55%. Of the last 4 patients, 3 were scheduled for chemotherapy with a change in 2 patients, and 1 patient for combined ADT and radiotherapy that was not changed.

**Table 3:** Impact on patient management: Treatment scheduled before and then in view of FCH PET/CT.

Management		Scheduled									
		ADT (n = 107)		Surgery (n = 5)		Radiotherapy (n = 12)		Watchful waiting (n = 51)		Other (n = 4)	
		Nb of patients	Result of FCH PET/CT	Nb of patients	Result of FCH PET/CT	Nb of patients	Result of FCH PET/CT	Nb of patients	Result of FCH PET/CT	Nb of patients	Result of FCH PET/CT
**Indicated in view of FCH PET/CT**	**ADT (n = 85)**	51	27+, 7?, 17-	4	3+, 1-	5	5+	25	20+, 2?, 3-	-	
	**Surgery (n = 8)**	6	6 +	-		-		2	1+, 1-	-	
	**Radiotherapy (n = 25)**	22	14+, 1?, 7-	-		3	1+, 1?, 1-	-		-	
	**Watchful waiting (n = 36)**	11	6+, 2?, 3-	-		2	2-	23	3+, 5?, 15-	-	
	**Other (n = 25)**									2^§^, 2*	2^§^+, 2*-
	HIFU	7	6+, 1-	-		-		-			
	cryotherapy	-		1	1?	-		-			
	ADT and surgery	2	2+	-		-		-			
	ADT and radiotherapy	2	2+	-		1	1+	-			
	ADT and brachytherapy	1	1+	-		-		-			
	lung surgery	-		-		-		1	1+		
	radiotherapy on prostate bed + lung surgery	1	1+	-		-		-			
	change in ADT	4	2+, 1?, 1-	-		-		-			
	change in radiotherapy planning	-		-		1	1+	-			

^+^ positive,

^?^ equivocal,

^-^ negative FCH PET/CT by patient-based on-site reading

* no change in 2 patients: chemotherapy alone in 1 patient, combined ADT and radiotherapy in 1 patient.

^§^ change in 2 patients: ADT added to scheduled chemotherapy in 1 patient, scheduled chemotherapy replaced by ADT in 1 patient.

When FCH PET/CT resulted in a management change, 45 patients were treated with curative intent (salvage radiotherapy in 26 cases including 1 with added ADT, surgery for prostate cancer in 10 cases and for a second cancer of the lung in 2 cases, HIFU in 7 cases, cryotherapy and brachytherapy in 1 case each).

According to type of previous treatment(s), the impact on patient management was the most frequently observed in patients treated by RP alone, by RP and ADT and by RP and EBRT.

The frequency of impact on patient management according to previous treatment(s) is summarised in Table 4. The impact on patient management has been observed the most frequently in patients after radical prostatectomy in whom the external beam radiation therapy could be indicated in place of medical treatment in 13/31 cases.

**Table 4:** Frequency of impact on patient management according to previous treatment(s).

Previous treatment	Frequency of impact in patient management % (and 95%CI if denominator >10)
RP	72% (31/43) (CI 56–85%)
EBRT, ADT	58% (23/40) (CI 41–73%)
RP, EBRT	50% (12/24) (CI 29–71%)
EBRT	29% (6/21) (CI 11–52%)
RP, ADT, EBRT	41% (7/17) (CI 18–67)
Brachytherapy	67% (8/12) (CI 35–90%)
HIFU	86% (6/7)
Brachytherapy, ADT	100% (3/3)
EBRT, ADT, CHT	33% (1/3)
RP, ADT, CHT	0% (0/3)
HIFU, EBRT	100% (2/2)
HIFU, ADT	0% (0/2)
EBRT, ADT, cryotherapy	100% (1/1)
RP, CHT	0% (0/1)

RP: radical prostatectomy, EBRT: external beam radiation therapy, ADT: androgen deprivation therapy, HIFU: high intensity focused ultrasound, CHT: chemotherapy

At FCH PET/CT, 13 patients were treated by ADT, the impact on patient management was observed in 7 of them (ADT was replaced by HIFU in 3 cases, continued ADT in place of scheduled watchful waiting in 2 cases, modification of ADT in 1 case and addition of pelvic lymphadenectomy to ADT in 1 case).

FCH PET/CT had no clinical impact in 79 patients (45%), 31 with positive, 13 with equivocal and 35 with negative FCH PET/CT. Of the 31 patients with positive FCH PET/CT and no change in management, radiotherapy was performed as planned in 2, planned watchful waiting was continued in 3 including 1 who refused salvage radiotherapy, and scheduled ADT was maintained in 27 patients in view of the spread of the disease on FCH PET/CT.

Forty-eight patients were in their second recurrence of PCa. Their PSA serum levels were not statistically different from those of patients on their first recurrence. In these 48 patients, FCH PET/CT yielded a positive result in 24 (50%), and a negative result in 24 cases (50%). As a consequence, the treatment with curative intent was indicated in 5 patients (10%) and palliative treatment in 13 patients (27%).

There was no significant difference in PSA serum levels, PSA doubling time or PSA velocity whether or not FCH PET/CT had a clinical impact. Median PSA level was significantly lower (p<0.0001) at the end of a 6 month follow-up period (0.5ng/mL, range non-detectable (ND)—447) than at the time of FCH PET/CT (3.6ng/mL, range 0.5–244).

After the follow-up period, the PSA median serum level was 0.31ng/mL (range ND-447) vs. 1ng/mL (range ND-16), respectively whether FCH PET/CT was positive or non-positive, and 0.3ng/mL (range ND-86) vs. 0.8ng/mL (range ND-447), respectively whether or not FCH PET/CT had a clinical impact, no difference being statistically significant.

### Adequacy of management changes motivated by result of FCH PET/CT

Of 100 patients for whom FCH PET/CT had a clinical impact, the changes were considered by the independent assessor as adequate in 89 cases (89%, CI = 71–100%). They were considered as inadequate in 11 cases: 4 patients had an unnecessary biopsy (false-positive FCH PET/CT findings, reported as only equivocal in 1 case), in 3 patients, treatment with curative intent indicated on the base of negative result of FCH PET/CT did not lead to decrease in serum PSA level (radiotherapy in 2 cases and surgery in 1 case) and a rapid increase of PSA serum level occurred in 4 patients after watchful waiting was decided in view of a negative result of FCH PET/CT.

## Discussion

To the best of our knowledge, this is the first multicentre prospective study that analysed the positivity rate, the clinical impact, and the adequacy of therapeutic decisions in view of FCH PET/CT in patients with occult biochemical recurrence of PCa.

### Positivity rate

In our series, we found a lower positivity rate for FCH PET/CT (59% if equivocal results were considered as negative) than in the previously published studies aiming to determine its impact on management in biochemical recurrence of PCa: 79% in the study by Soyka et al. [14] and 80% in the recently published study by Colombié et al. [15]. This might be explained by our inclusion criteria restricted to patients with an occult biochemical recurrence of PCa with negative or equivocal results of imaging modalities, at least pelvic MRI and bone scintigraphy. In the study of Soyka et al. the results of others imaging modalities were not a part of inclusion criteria [14]. Furthermore, no equivocal result of FCH PET/CT was taken into account in those two studies; in our series, considering equivocal results as positive raises the positivity rate to 70%.

In agreement with other studies, we found that positivity of FCH PET/CT was linked with PSA serum level at FCH PET/CT [7, 13–17] and with PSA velocity [16] but not with the Gleason score [15], although this relation has been reported by Cimitan et al. in a large series [18].

Thus, a lower mean PSA serum level in our series (7.5 ng/mL) than in that of Colombié et al. (10.7 ng/mL) [15] may have contributed to the lower positivity rate in our series. In recent retrospective studies that did not address clinical impact, a lower positivity rate of 45.6% has been reported in a multicentre Spanish study with a mean PSA level of 5.3 ng/mL [17], and a positivity rate of 58.2%, similar to ours, in another large series with a mean PSA level of 5.5 ng/mL [8].

### Clinical impact

In our series, FCH PET/CTs had a clinical impact in 56% of patients, significantly more frequently in case of positive result. This rate is slightly higher than those reported in the previous retrospective studies that addressed this objective in an identified group of patients with biochemical recurrence of PCa (Table 5): 48% [14], 43.6% [15]. Using ^11^C-choline (in all or in a large majority of patients) instead of the fluorinated analogue FCH, very concordant impact rates of 46.7% [19] and 54.5% [20] have been reported in this setting.

**Table 5:** Clinical impact of FCH PET/CT in biochemical recurrence of prostate cancer: Comparison and pooling with the results of other studies (An impact corresponds to a change in patient management induced by the results of FCH PET/CT; percentage values have been rounded).

Reference	Nb of patients	Nb of centre(s)	Type of study	Others imaging modalities	Positivity rate	Impact rate	Adequacy rate of induced changes
**Present study**	179	15	Prospective	Negative or inconclusive	59%	56%	89%
**Soyka et al. [14]**	156	1	Retrospective	Not considered	79%	48%	Not evaluated
**Colombié et al. [15]**	172	1	Retrospective	Negative or inconclusive	80%	44%	Not evaluated
**OVERALL**	**507**	**17**			**72%**	**49%**	
**95% CI** (ignoring differences in methodology)					**65–80%**	**43–56%**	

Thanks to the prospective design of the present study, the method to estimate the clinical impact of FCH PET/CT was different. The questionnaire concerning the scheduled management had to be filled out twice by the referring physician, before and after FCH PET/CT. Soyka et al. [14] evaluated the clinical impact retrospectively on basis of one single questionnaire sent retrospectively to the referring physician. A similar “hypothetical” approach was used by Colombié et al. [15].

Only Colombié et al. [15] differentiated the rate of clinical impact in case of non-positive FCH PET/CT: 20% vs. 35% (26/74) in our study. It can be concluded that even a negative FCH PET/CT can have a clinical impact in this setting.

Overall, our results confirm the significant impact of FCH PET/CT on PCa management in case of biochemical recurrence. By pooling the results of the 3 studies, although there were significant differences in their methodology as discussed above, the overall impact rate is 49% with a rather narrow 95% confidence interval: 43–56% (Table 4).

Several studies showed that salvage radiation therapy in PCa recurrence was feasible, well tolerated and could defer ADT for several months [21, 22]. A multicentre phase II trial is currently being performed to investigate the metastasis directed therapy by surgery or salvage radiation therapy in this setting [22]. But the selection of patient is a crucial matter to avoid unnecessary treatment and radiation-induced morbidity. In our study, 43% of patients in whom FCH PET/CT showed distant metastases were previously scheduled for curative treatment; conversely, 38% of patients scheduled for ADT finally received a curative treatment. Those results show that FCH PET/CT provides an additional value to select the patients who are eligible for a curative treatment.

### Adequacy of therapeutic decisions

After the 6 month follow-up period, the PSA serum levels were not significantly different whether or not FCH PET/CT was positive or had a clinical impact. Soyka et al. [14] found the same results. Most patients received ADT alone or in combined therapy (ca. 55%—Table 3) and a decrease in PSA serum level was then expected. Thus a decrease in PSA serum level 6 months after the management decision did not appear to be a relevant indicator of the adequacy of the management decision based on imaging.

In our study, 89% of changes in management motivated by results of FCH PET/CT were evaluated by an independent assessor as adequate. This analysis has not been done in previous studies; actually the result would have been uncertain due to their retrospective design.

Radiolabelled choline as a tracer of lipid metabolism is not a specific ligand for prostate cancer [23–25]. In our study, thanks to whole-body character of FCH PET/CT imaging, two lung cancers and two thyroid nodules could be diagnosed; only the lung nodules were further characterised. FCH uptake by lung cancer [24, 25] or by benign [23, 26, 27] or malignant [26] thyroid nodules has already been reported. Thus an adequate decision based on FCH PET/CT in case of biochemical recurrence of prostate cancer requires a careful evaluation of the whole-body and to prompt a complementary evaluation of equivocal distant foci, since the therapeutic approach and the prognosis may be completely modified by finding a metachronous cancer instead of a distant metastasis of prostate cancer.

## Limitations

The main limitations of the study are related to the selection criteria. In order to obtain a significant proportion of positive FCH PET/CT, the biochemical inclusion criterion was aimed to select a population with a relatively active PCa recurrence, not just PSA serum level > 0.5 ng/mL. Consequently, we might expect a lesser positive rate and clinical impact in a general population with early biochemical recurrence of PCa. Also the lack of long-term outcome data does not enable us to demonstrate a correlation between the results of FCH PET/CT and overall patient survival. Studies aiming at the assessment of long term outcome of patients who underwent FCH PET/CT due to biochemical recurrence of PCa are lacking because of the rather longer life expectancy of PCa at that stage, which requires a follow-up over 5 years at least. Furthermore, during such long time frame, the performance of the PET/CT equipment will probably be improved, making the results of such a study of uncertain significance.

Another limitation, common to most studies in recurrent prostate cancer, is the lack of a standard of truth that can be determined for all patients and all sites. This was due to the well-known fact that histology of foci after re-intervention of biopsy can only be obtained in a limited number of cases. A large part of the patients were treated with hormone therapy leading to a decrease in PSA serum levels but without possibility of confirming the FCH foci as true-positive results.

## Conclusion

This first multicentre prospective study shows that FCH PET/CT is an effective tool with substantial impact on therapeutic management in patients with occult biochemical recurrence of prostate cancer.

## Supporting information

S1 Table The example of second questionnaire concerning the actual patient’s therapeutic management after FCH PET/CT. (PDF)
