# Supplementary material for: 18F-fluorocholine PET/CT in patients with occult biochemical recurrence of prostate cancer: Detection rate, impact on management and adequacy of impact. A prospective multicentre study
Source: PLoS One. 2018 Feb 9;13(2):e0191487. doi: 10.1371/journal.pone.0191487 (PMC5806856; doi:10.1371/journal.pone.0191487)
Supplement: S1 Table — (PDF) [file pone.0191487.s001.pdf]

S1 Table. The example of second questionnaire concerning the actual patient’s therapeutic management after FCH PET/CT.

IDENTIFICATION DU PATIENT			
**SITE**	**PATIENT N°**	**INITIALES PATIENT**	**DATE D’INCLUSION**
**Date de l’examen TEP/TDM:**			
**ETAT APRES L’EXAMEN TEP/TDM LORSQUE LA DECISION THERAPEUTIQUE A ETE PRISE (ADRESSER AU CLINICIEN DEMANDEUR DANS LES 2 MOIS)**			
**Répondre aux questions, en entourant la réponse correspondant au cas du patient**			
1) L’examen TEP/TDM vous a-t-il fait pratiquer un ou plusieurs autres examens de confirmation ?			
a) Oui			
b) Non			
2) Si vous avez répondu OUI à la question 2), estimez-vous que ces examens ont été profitables pour le patient ?			
a) Oui			
b) Non			
3) L’examen TEP/TDM (éventuellement suivi des examens de confirmation) a-t-il modifié l’attitude thérapeutique ou la prise en charge du patient ?			
a) Non			
b) Chirurgie remplacée par traitement médical			
c) Chirurgie remplacée par radiothérapie			
d) Chirurgie remplacée par abstention thérapeutique			
e) Traitement médical remplacé par chirurgie			
f) Traitement médical remplacé par radiothérapie			
g) Traitement médical remplacé par abstention thérapeutique			
h) Radiothérapie remplacée par traitement médical			
i) Radiothérapie remplacée par chirurgie			
j) Radiothérapie remplacée par abstention thérapeutique			
k) Abstention thérapeutique remplacée par traitement médical			
l) Abstention thérapeutique remplacée par chirurgie			
m) Abstention thérapeutique remplacée par radiothérapie			
n) Modification du traitement médical			
o) Modification du geste chirurgical			
p) Modification du protocole de radiothérapie			
q) Autre, préciser			
En cas de traitement médical, préciser s’il s’agit de chimiothérapie, hormonothérapie, ou de l’association des deux:			
**Date :**			
**Nom** en majuscules et signature du médecin demandeur qui a rempli le formulaire :			
**Signature :**			
